# RETRACTION: The influence of Chinese Herbal Medicines on Cancer‐Related Pressure Ulcer Wound, Fatigue, Constipation, and Anorexia: A Meta‐Analysis

**DOI:** 10.1111/iwj.70422

**Published:** 2025-03-26

## Abstract

**RETRACTION:** LiH. and LiuH. , “The influence of Chinese Herbal Medicines on Cancer‐Related Pressure Ulcer Wound, Fatigue, Constipation, and Anorexia: A Meta‐Analysis,” International Wound Journal 20, no. 1 (2023): 28–37, 10.1111/iwj.13833.35582926 PMC9797920

The above article, published online on 18 May 2022, in Wiley Online Library (http://onlinelibrary.wiley.com/), has been retracted by agreement between the journal Editor in Chief, Professor Keith Harding; and John Wiley & Sons Ltd. Following an investigation by the publisher, all parties have concluded that this article was accepted solely on the basis of a compromised peer review process. The editors have therefore decided to retract the article. The authors did not respond to our notice regarding the retraction.

**RETRACTION:** H. Li and H. Liu , “The influence of Chinese Herbal Medicines on Cancer‐Related Pressure Ulcer Wound, Fatigue, Constipation, and Anorexia: A Meta‐Analysis,” International Wound Journal 20, no. 1 (2023): 28–37, 10.1111/iwj.13833.35582926 PMC9797920

The above article, published online on 18 May 2022, in Wiley Online Library (http://onlinelibrary.wiley.com/), has been retracted by agreement between the journal Editor in Chief, Professor Keith Harding; and John Wiley & Sons Ltd. Following an investigation by the publisher, all parties have concluded that this article was accepted solely on the basis of a compromised peer review process. The editors have therefore decided to retract the article. The authors did not respond to our notice regarding the retraction.

